# Digital Tools for the Promotion of Healthy and Sustainable Eating Behaviors in the General Population: A Systematic Review of the Literature

**DOI:** 10.3390/nu18040645

**Published:** 2026-02-16

**Authors:** Valentina Gardini, Marco Luis Paolillo Diodati, Cristina Mori, Elena Tomba

**Affiliations:** Department of Psychology, University of Bologna, 40127 Bologna, Italy; valentina.gardini8@unibo.it (V.G.); marcoluis.paolillo@studio.unibo.it (M.L.P.D.); cristina.mori@unibo.it (C.M.)

**Keywords:** healthy eating, sustainable eating, digital interventions, food sustainability, psychological interventions

## Abstract

**Background/Objectives**: Promoting healthy and sustainable food choices is critical to address environmental and public health challenges, prevent health issues and enhance psychological well-being. Technological tools have shown promising results in supporting the adoption of many sustainable practices and in improving dysfunctional eating behaviors in clinical psychological settings. However, their potential to encourage healthy and sustainable eating choices in the general population through psychological or behavioral strategies remains understudied and unsystematically observed. **Methods**: A systematic review was conducted following PRISMA guidelines to (1) investigate digital tools and interventions aimed at improving healthy and sustainable eating behaviors, and (2) categorize the psychological or behavioral strategies they implemented. Four databases (PsycINFO, PsycArticles, PubMed, ProQuest) were searched combining keywords on sustainable diets (e.g., “sustainable diet,” “food sustainability”) and technological tools (e.g., “virtual reality,” “mobile app,” “web app”). **Results**: N = 16 studies were included. N = 7 (44%) used mobile app-based tools, *n* = 6 (38%) were virtual reality, *n* = 2 (12%) were web platforms, and *n* = 1 (6%) was an instant-messaging system. Digital tools and interventions were useful in promoting healthy and sustainable eating behaviors by implementing psychological or behavioral strategies like awareness (*n* = 10, 63%), decision-making (*n* = 6, 38%), emotion regulation (*n* = 3, 19%), nudging (*n* = 5, 31%), self-efficacy (*n* = 5, 31%) and self-monitoring (*n* = 4, 25%). Only a few studies included follow-ups (*n* = 5, 31%). **Conclusions**: Findings suggest that digital technologies have the potential to improve healthy and sustainable eating behaviors in the general population. However, given heterogeneity and methodological issues of studies, more longitudinal and rigorous research is needed to confirm the effectiveness and long-term benefits of different technological tools.

## 1. Introduction

The increasing need to address global environmental and health challenges has made it essential to explore innovative strategies for promoting healthy and sustainable eating.

Indeed, food systems play a central role in both public health and environmental sustainability. More specifically, dietary patterns characterized by high consumption of animal-based and ultra-processed foods have been associated with an increased risk of chronic diseases, including cardiovascular diseases and metabolic disorders [1,2], as well as with higher greenhouse gas emissions, land use, and water consumption [3]. Moreover, a growing body of scientific evidence also suggests that poor diet quality in children and adolescents is associated with deteriorating mental health [4], and that a balanced diet can reduce the risk of developing psychological disorders such as anxiety and depression [5]. Conversely, a bidirectional relationship emerged between mental health and diet: mental disorders, particularly eating disorders, can lead to dysfunctional and environmentally damaging eating behaviors [6], which in turn can negatively impact both physical and psychological well-being [7].

The One Health approach, defined by the World Health Organization, is a novel paradigm stating that the health of humans, animals, and ecosystems are closely interlinked and interdependent [1], and essential for transitioning towards more sustainable food systems [8]. This paradigm supports the ways that dietary patterns impact not only public and individual health but also the environment and the welfare of animals in the context of food systems. According to recent modeling studies, dietary changes toward plant-based and flexitarian (that is, having a primarily vegetarian diet but occasionally eating meat or fish) patterns can have a positive impact on human health outcomes by 25%, reduce greenhouse gas emissions by up to 55%, and reduce animal welfare loss by 52–97% depending on the dietary pattern [9]. A strong justification for the adoption and promotion of sustainable diets as part of an integrated health strategy is provided by this systems-level framing, which aids in bringing environmental sustainability and public health objectives together.

This body of empirical and theoretical evidence highlights how the promotion of sustainable eating habits could improve both the environmental impact of the food system and an individual’s physical and mental health [10], and therefore it needs an integrated perspective. However, the promotion of sustainable eating should not overlook the fact that not all sustainability-oriented dietary practices are inherently health-promoting if they are highly restrictive or nutritionally unbalanced [11,12,13,14]. Indeed, international guidelines such as those proposed by the Food and Agriculture Organization (FAO) [15] emphasize that healthy and sustainable diets should simultaneously ensure nutritional adequacy, low environmental impact, and positive health outcomes. Since these dimensions are not always jointly operationalized in empirical research, studies often focus on isolated aspects of healthy and sustainable eating (e.g., considering only meat reduction or food waste), leading to heterogeneity and difficulties in comparing intervention outcomes across studies.

Another issue that has emerged in the literature is that behavioral changes required to adopt healthy and sustainable eating habits are often hindered by cognitive, social, and cultural barriers. These include strong beliefs about meat consumption (e.g., perceiving meat as healthy, necessary, or socially normative) and emotional attachment to traditional dietary patterns [16,17,18], a lack of environmental concern [19], a lack of perceived personal relevance together with attachment to habitual food practices [20], as well as structural and commercial barriers (e.g., limited availability, convenience, or variety of sustainable food options) [21,22].

In this context, technology could represent a promising tool for overcoming these barriers by providing innovative tools to inform and engage consumers towards more healthy and sustainable practices [23]. From smartphone applications to web platforms and virtual reality (VR), digital technologies have proven effective in positively influencing sustainable behaviors, increasing awareness, and making sustainability-related information more accessible [24]. Some of these tools leverage psychological principles to facilitate behavioral change through mechanisms such as gamification and nudging, guiding users toward more conscious and sustainable choices [25]. In addition, Design for Sustainable Behavior (DfSB) is an emerging research area that uses smart devices and persuasive technologies to change consumer behavior, promoting more sustainable actions [26]. Another most frequently employed theory to encourage sustainable behaviors is the Theory of Planned Behavior (TPB) [27], which suggests that a person’s behavior is influenced by his or her intentions, which in turn are shaped by attitudes, subjective norms and perceived behavioral control [28].

In terms of eating behaviors, digital technologies and interactive platforms have emerged as useful tools in clinical psychology as well, where they have been widely and successfully used in the treatment and prevention of eating disorders (EDs), supporting clinical interventions and improving the management of such conditions [29,30,31], as well as prevention [32,33,34].

Despite the body of evidence suggesting that digital technologies can be used to promote healthy eating habits, for example by reducing dysfunctional eating behaviors and preventing EDs, their specific impact on the adoption of sustainable and healthy eating habits has not yet been systematically investigated. Moreover, while previous studies have examined specific sustainable eating behaviors (e.g., meat reduction or food waste) [23,35,36,37], there is still a lack of systematic syntheses on which specific psychological and behavioral strategies have been implemented across different digital interventions to promote healthy and sustainable eating. Therefore, this systematic narrative review of the literature has two complementary aims: (1) to explore and synthesize how emerging digital technologies can promote sustainable food choices in the general population, in order to help identify which digital tools have been more frequently used in the literature to improve healthy and sustainable food choices and their effects; and (2) to explore and systematically categorize which psychological and behavioral strategies have been implemented by these technological tools to promote healthy and sustainable eating behaviors.

## 2. Materials and Methods

### 2.1. Search Strategy

A systematic narrative literature search was conducted following the PRISMA (Preferred Reporting Items for Systematic Reviews and Meta-Analyses) guidelines [38]. Four electronic databases were systematically queried: PsycInfo, PsycArticles, PubMed, and ProQuest. The search strategy combined terms related to sustainable dietary behaviors with terms referring to digital and immersive technologies. The selection of search terms was guided by the recognition that the literature currently lacks a standardized and universally accepted set of terms to define healthy and sustainable eating behaviors. Therefore, a broad and inclusive set of keywords was employed to capture dietary patterns, environmental sustainability dimensions, and healthy food-related behaviors (e.g., not unbalanced or overly restrictive). This approach was adopted to maximize the identification of digital interventions targeting healthy and sustainable eating behaviors across different conceptualizations.

The Boolean search string used was:

(“sustainable diet” OR “sustainable eating” OR “sustainable food choices” OR “sustainable eating behavior” OR “food sustainability” OR “ethical eating” OR “plant-based diet” OR “meat reduction” OR “food waste reduction” OR “climate-friendly diet” OR “ecological footprint of diet” OR “locally sourced food” OR “seasonal eating” OR “fair trade”) AND (“virtual reality” OR “mobile app” OR “artificial intelligence” OR “augmented reality” OR “web app” OR “AR/VR platforms” OR “AI applications” OR “AR/VR experiences” OR “machine learning” OR “AR/VR” OR “AI in healthcare”).

Given the relatively recent integration of these technologies in the context of clinical psychology and sustainable nutrition, no publication date restrictions were applied. Filters were applied to include only peer-reviewed empirical studies, and to exclude dissertations, essays, books, book chapters, reviews, commentaries, and conference proceedings.

Duplicate articles were removed as the first step in the review process. Thereafter, titles and abstracts were screened by two authors (Author 2 and Author 3). Articles that did not meet the inclusion criteria and that investigating variables not relevant to the research topic were excluded. The same two authors then independently assessed the full texts of relevant studies for the review. In case of disagreement, the full texts were revised and discussed by a third author (Author 4) until consensus was reached by all authors. Data from the selected articles were then extracted and summarized by two authors (Author 1 and Author 2). This systematic narrative review protocol was registered in PROSPERO (ID: CRD420250651036). The literature search was conducted in March 2025, and the systematic review process was concluded in August 2025.

### 2.2. Eligibility Criteria

Inclusion and exclusion criteria were defined according to the PICOS framework (Population, Intervention, Comparison, Outcome, Study Design) [39], as detailed in Table 1 below. Eligible studies were those reporting empirical data on the use of digital or immersive technologies (e.g., AR/VR, mobile applications, AI-based tools) to promote sustainable dietary practices. Studies were excluded if they (a) did not involve human participants, (b) did not describe an implemented methodology, or (c) were purely conceptual or theoretical in nature.

The initial search yielded 10 records from PsycInfo, 1 from PsycArticles, 12 from PubMed, and 8 from ProQuest, for a total of *n* = 29 unique articles after removing *n* = 2 duplicates. Based on title and abstract review, *n* = 10 articles were removed.

The remaining *n* = 20 articles were then retrieved and assessed for eligibility through full-text examination. This led to the exclusion of *n* = 2 studies where no digital tool was used, *n* = 2 protocol papers without experimental data, and *n* = 2 about the design and development digital tools that did not report statistical data about their effects on eating behaviors. After the addition of *n* = 2 articles found through manual search, a total of *n* = 16 articles was included in the review.

**Figure 1:**
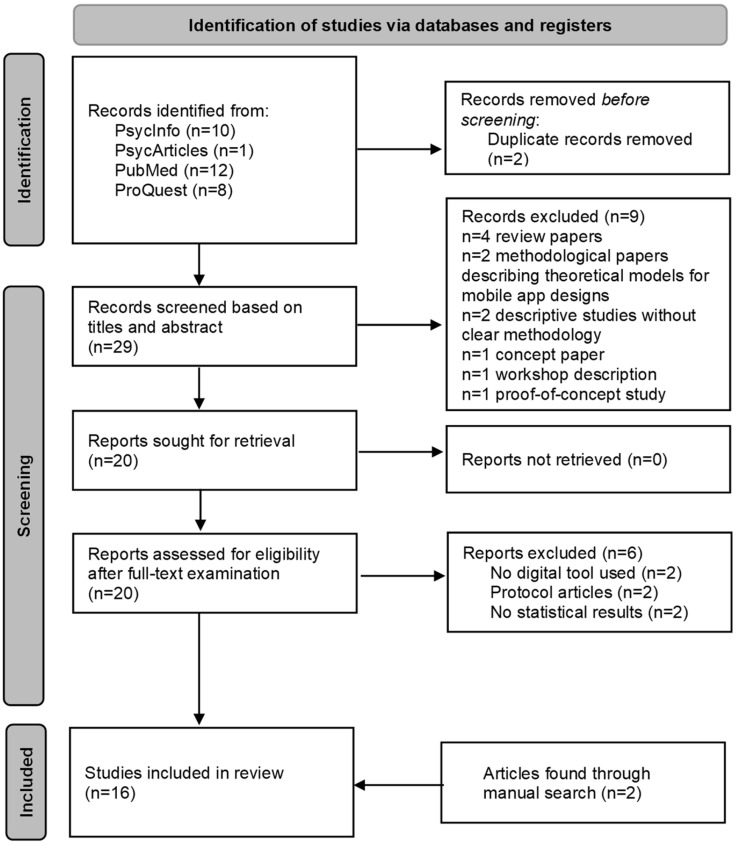
Flowchart showing the process of selecting articles for inclusion in the review according to the PRISMA criteria.

The results of the literature search process are summarized in Figure 1.

Subsequently, in order to understand which psychological and/or behavioral strategies were implemented by the technological tools used in the studies, these strategies were identified using an inductive qualitative coding approach. In particular, when explicit labels identifying strategies were not provided by authors of the selected studies, strategies were inferred based on how interventions were designed, which processes they appeared to target and/or the reported outcomes. During the analysis, the identified strategies were subsequently categorized according to established definitions to ensure conceptual coherence and consistency across studies. The identification and categorization process was conducted independently by two authors (Author 1 and Author 2), and any discrepancies were resolved through discussion until consensus was reached. Strategies emerging from this process have been summarized in Section 3.4.2.

### 2.3. Quality and Risk of Bias Assessment

Studies included were then evaluated by two authors (Author 1 and Author 2) using a customized checklist adapted from the National Institutes of Mental Health’s tools [40], which allows for an overall assessment of the quality. The methodological quality of the studies was evaluated on three levels: strong, moderate or weak (criteria and scores for each item have been provided and summarized in Table S2). In order to establish the quality of the study, it was necessary for the reviewers to reach a consensus by comparing the ratings and identifying any areas of disagreement. Differences in the study quality ratings were addressed through discussion of each item on the rating checklist (see Table S3).

## 3. Results

### 3.1. Characteristics of the Studies

Characteristics and main results of the studies included in the systematic narrative review are summarized in Supplementary Table S1.

The randomized controlled trial (RCT) represents the most common experimental design among the selected studies (*n* = 9/16, 56%) [35,36,41,42,43,44,45,46], while *n* = 8 (50%) were non-controlled studies [37,47,48,49,50,51,52,53]. However, 31% (*n* = 5; *n* = 4 RCTs [36,42,43,45] and *n* = 1 non-controlled study [47]) included follow-up (FU) assessments; in four of them (*n* = 4/5, 80%) the FU ranged from 1 to 4 weeks [36,42,43,45], and in one (*n* = 1/5, 20%) the FU spanned 8 to 11 weeks [47].

The sample size across the selected studies varies substantially. The majority (63%, *n* = 10/16) included over 100 participants [36,37,41,42,43,45,46,48,49,50,51], with six of these (*n* = 5/10, 50%) having more than 200 participants [41,42,43,46,51]. A smaller portion (19%, *n* = 3/16) involved samples between 50 and 100 participants [35,44,52], while the remaining 19% (*n* = 3/16) included less than 50 participants [47,53,54].

Most studies employed samples of healthy, non-vegetarian adults. Specifically, 63% (*n* = 10/16) of the selected studies included only healthy, normal-weight adults [35,37,41,42,46,47,50,52,53]. Academic contexts were common: 31% of studies (*n* = 5/16) focused on university students, with mean ages ranging from 18.9 (SD = 1.1) to 25.03 (SD = 6.4) years old [36,43,45,51,54]. Only one study (*n* = 1, 6%) involved middle school students (M = 14.3 years, SD = 0.6) [44], and one (*n* = 1, 6%) included people with obesity (ages 19–64, Body Mass Index ≥ 25.0 kg/m^2^) [48].

Regarding gender, 94% (*n* = 15/16) of studies included mixed-gender samples [35,36,37,41,42,43,44,46,48,49,50,51,52,53,54], with two studies (13%) including participants identifying as “other” or “non-specified” gender [41,44]. One study (6%) focused exclusively on adult women [47].

### 3.2. Assessment Tools

Regarding assessment tools, the majority of studies (69%, *n* = 11/16) relied on ad hoc questionnaires or interviews [35,36,41,42,43,44,45,46,47,48,52], although only one (*n* = 1, 6%) [36] explicitly referenced the original Theory of Planned Behavior Questionnaire. Some studies incorporated additional tools, such as Bland–Altman plots and usability interviews (*n* = 1, 6%) [48], online food diaries (*n* = 1, 6%) [36], or semi-structured interviews and observations (*n* = 1, 6%) [47]. Only 29% (*n* = 5/16) used standardized psychometric scales [37,50,51,53,54].

### 3.3. Quality and Risk of Bias Assessment Results

Following the review process, the selected articles were assessed for quality. A total of 44% (*n* = 7/16) of studies were rated as strong [36,41,43,44,45,46,51], 25% (*n* = 4/16) as moderate [35,42,50,52], and 31% (*n* = 5/17) as weak [37,47,48,53,54] (see Supplementary Table S3). Weak ratings were most frequently driven by limitations related to FU duration. In particular, all studies lacked long-term FUs, with either no FU (*n* = 9, 53%) [35,37,44,46,48,51,52,53,54] or shorter than three months (*n* = 7, 44%) [36,41,42,43,45,47,50]. Moreover, methodological issues that decreased quality of the selected studies also included small sample sizes (*n* = 4, 25%) [47,48,53,54] and risk of bias, specifically selection bias (*n* = 6, 38%) [35,37,43,47,48,54] and attrition bias (*n* = 7, 44%) [41,42,44,48,49,50,54]. Most studies (*n* = 9, 56%) also did not control for confounding variables [36,37,42,47,48,50,51,53,54].

### 3.4. Results of the Selected Studies

The results of the studies selected for the current systematic narrative review will be reported in subsequent sections according to the two aims of the review. In particular, Section 3.4.1 will present results about the effects of digital technologies on healthy and sustainable eating behaviors (aim 1), whereas Section 3.4.2 will describe and categorize psychological and behavioral strategies through which these digital tools aimed to promote eating behavior change (aim 2).

#### 3.4.1. Statistical Results by Type of Technology

The first aim of this systematic narrative review was to evaluate the effects of technology in promoting healthy and sustainable eating behaviors. The selected studies show high heterogeneity in the types of technology employed. Specifically, 38% (*n* = 6/16) used virtual reality (VR) interventions [35,43,44,45,52,54], while 44% (*n* = 7/16) implemented mobile app-based tools [37,41,42,46,47,48,49,50]. Among the remaining studies, two focused on web-based platforms (*n* = 2, 12%) [51,53], and one used WhatsApp messaging (*n* = 1, 6%) [36].

##### Virtual Reality

Virtual reality (VR) was used in 38% of studies (*n* = 6/16). Results of VR-based interventions were promising in increasing healthy and sustainable food choices (F(4, 241) = 1680, *p* < 0.001, r^2^ = 0.20 [43]), reducing dietary footprint (d = 0.4, *p* = 0.034 [45]), and increasing awareness of the consequences of food choices (*p* < 0.001 [45]; *p* = 0.028 [44]). One study [54] also simulated a VR restaurant and found that VR messages (i.e., warning message of an animal hurting from plastic exposure) had a significant influence on the choice of more sustainable meals (*p* = 0.04), but not on packaging selection. The study also found higher sustainable behavior scores in participants selecting vegetarian meals (M = 6.4) over meat/fish (M = 5.7).

VR also increased presence (*p* = 0.039), empathy (*p* < 0.001), and intentions to reduce meat consumption (*p* = 0.022) in one study where participants were exposed to a VR documentary on slaughterhouse scandals [35], while another [52] demonstrated that red tables (vs. green) in a VR environment reduced meat selection (*p* = 0.0038, d = 0.34).

##### Mobile Apps

Mobile apps (*n* = 7/16; 44%) were useful in disrupting old habits and promoting mindful food choices [37,41,42,46,47,48,50]. One study by Carfora & Catellani (2023) [42] found that, when using a mobile app that sent notifications to participants, combining informative messages with dynamic norms (i.e., social norms communicated by emphasizing that an increasing number of people are adopting a given behavior over time) reduced meat and increased legume consumption (*p* < 0.05). Another study by Carfora et al. (2024) [41] showed that regulatory fit in persuasive messages (i.e., alignment between message framing and individuals’ motivational orientation that increases persuasiveness) boosted attitude (F = 6.53, *p* = 0.011), desire (F = 7.36, *p* = 0.007), intention (F = 4.64, *p* = 0.032), and actual downloads (χ^2^ = 5.21, *p* = 0.022) of a mobile app that promotes sustainable eating.

The authors of [46] also demonstrated that eco-ranking systems in a mobile app (i.e., showing participants the ecological score associated with food products that could be found in a supermarket) promoted sustainable eating choices (*p* < 0.001).

Regarding qualitative studies, four app-based studies lacked clear statistical outcomes, as their data were more qualitative and descriptive, and so less robust in terms of measuring the real effects of the instruments [37,47,48,50]. Specifically, Flaherty et al. (2020) [47] found that participants reported that their mobile app (which allowed individuals to set their goals and provided a daily menu and weekly shopping lists with healthier food choices) facilitated the breaking of existing habits and promoted a more conscious and reflective approach to the decision to purchase food. Similarly, De Croon et al. (2025) [50] found that their food recommender app produced healthier and more sustainable dietary changes in participants, including higher consumption of vegetables and fruits compared to carbohydrates, reduced consumption of snacks and sweets, decreased reliance on dairy products, and increased contribution of vegetables in total protein for some participants. Another mobile app [48] was evaluated by women with obesity as easy, simple, attractive, and informative in monitoring calories, dietary composition and providing nutritional information, thus helping healthier and more sustainable food choices. Finally, Haas et al. (2022) [37] instead showed that their mobile app impacted 73% of participants to become more aware of their food waste.

##### Web-Based Platforms

Two studies (*n* = 2/16; 12%) employed web-based interventions. “The Green Hub” [53] (a modular program consisting of a variety of activities posted on their web-platform, including informative posts, quizzes, and cooking videos) demonstrated positive effects on healthy and sustainable dietary behavior (i.e., consumption of red meat from 40% to 26%, fast food from 20% to 0%, food waste from 7% to 0%, consumption of vegetable proteins from 86.7% to 100%, and purchase of 0 km food from 73.4% to 100% and seasonal food from 83.3% to 100%). However, findings are limited by the absence of a control group, a small sample size, and the lack of statistical analyses measuring significance of changes.

In contrast, the similar but older web-based platform “The Green Eating Project” [51] yielded more methodologically robust results, with significant improvements in both sustainable eating behaviors (*p* < 0.001, η^2^ = 0.03) and knowledge about food sustainability (*p* < 0.001, η^2^ = 0.11) compared to controls not using the platform.

##### Instant Messaging Systems (WhatsApp)

The study employing instant messaging systems (i.e., WhatsApp) [36] demonstrated significant effects in promoting more healthy and sustainable dietary behaviors through daily messages that reminded the participants to reduce their meat consumption for 1 week. Participants exposed to the intervention reduced their consumption of processed meat (M = 1.74 servings/week) compared to the control group (M = 3.29 servings/week; *p* < 0.001) and showed a significant increase in their intention to reduce processed meat intake (*p* < 0.008).

#### 3.4.2. Psychological Factors and Behavioral Strategies Implemented Through Technology to Improve Healthy and Sustainable Eating Behaviors

The second aim of this systematic narrative review was to explore and systematically categorize which psychological and behavioral strategies have been implemented by the found digital tools to promote healthy and sustainable eating behaviors.

In the articles included in the review, *n* = 13/16 (81%) studies explicitly stated which psychological or behavioral strategies were implemented in the digital tools [36,37,41,43,44,45,46,47,48,50,52,53,54]. Of these, while in *n* = 3 studies [41,43,50] some psychological and behavioral strategies were explicitly stated by the original authors, a detailed full-text analysis led the authors of the present systematic narrative review to infer that additional strategies were also implemented within the design and functioning of the digital tools, even without them being explicitly stated. In *n* = 3 studies (19%), instead, strategies had to be fully inferred by the authors through the coding process described above [35,42,51].

This whole process led to the categorization of six main psychological or behavioral strategies used by the digital tools in the studies to increase healthy and sustainable eating behaviors:

- Awareness was identified for interventions aimed at increasing individuals’ knowledge, understanding, or perception of the health and/or environmental consequences of food-related behaviors (63%, *n* = 10/16).
- Decision-making was identified for interventions designed to support deliberation, comparison of options, or evaluation of consequences at the moment of food choice, thereby facilitating more informed and reflective decisions (38%, *n* = 6/16).
- Nudging was identified for interventions that modified the choice architecture to guide behavior in a predictable way without restricting options, for example through prompts, defaults, or subtle environmental cues (31%, *n* = 5/16).
- Self-efficacy was identified for interventions aiming to enhance individuals’ confidence in their ability to perform and maintain healthy and sustainable eating behaviors (31%, *n* = 5/16).
- Self-monitoring was identified for interventions that allowed individuals to track, record, or receive feedback on their eating behaviors, thereby supporting self-regulation and awareness of progress over time (25%, *n* = 4/16).
- Emotion regulation was identified for interventions aiming to influence emotional processes related to food choices, such as anticipated emotions (e.g., anticipated regret), emotional engagement, or affective responses that motivate behavior change (19%, *n* = 3/16).

##### Awareness

Awareness emerged as the most frequently used psychological strategy to improve healthy and sustainable eating behaviors, featured in 63% (*n* = 10/16) of the digital tools [35,37,42,44,45,50,51,53,54].

The qualitative study by Haas et al. (2022) [37] implemented a mobile app targeting food waste reduction using awareness about the topic. Following the use of the app, 73% of participants reported an increase in awareness measured via an ad hoc questionnaire. As mentioned above (Mobile Apps Section), participants also stated that, by increasing their awareness on the topic, the app also led to the reduction of food waste. Other previously described studies using mobile apps (Mobile Apps Section) in which awareness was implemented successfully managed to increase the consumption of legumes over meat [42], to encourage healthier dietary changes (e.g., increased intake from vegetables and fruits compared to carbohydrates in the studies by De Croon et al., 2025 [50]), and to influence consumers’ choice of more sustainable food options [46].

Several studies using VR mentioned in Virtual Reality Section used immersive environments to increase awareness by enhancing the visual impact of food choices and, therefore, increase healthy and sustainable eating behaviors. These studies showed that VR-based interventions using awareness strategies managed to reduce individuals’ footprints (d = 0.4, *p* = 0.034) [45] and reduce meat consumption (*p* = 0.022 [35]). Another VR study [54] found that infographics about ocean pollution led to more sustainable food and packaging choices (*p* = 0.04).

Finally, in *The Green Hub* study and *The Green Eat Project* (described in Web-Based Platforms Section), informative web-based content was also directed at increasing awareness on sustainable diets through interactive tools or personalized feedback. These studies reported both an increase in green eating knowledge (F(1, 407df) = 51.15, *p* <.001, η^2^ = 0.11) compared to controls who did not use the platform and healthy and sustainable eating behaviors.

##### Decision-Making

Decision-making was targeted in 38% (*n* = 6/16) of the selected studies [41,43,46,47,50,51]. More specifically, studies found digital tools could facilitate more informed and reflective decisions towards healthier and more sustainable food choices by supporting deliberation, comparison of options, or evaluation of consequences at the moment of food choice.

Starting from VR-based intervention, the study by Meijers et al. (2022) described above (Virtual Reality Section) [43] used informative messages about ingredients and the environmental impact of foods present in a VR environment to increase sustainable food choices (F(4, 241) = 16.80 *p* < 0.001, r^2^ = 0.20 compared to controls).

Decision-making was also facilitated in the aforementioned mobile apps through motivational messages that activated regulatory fit [41], and through personalized app feedback [50] in a qualitative study with high drop-out rates (30%). Another qualitative study on a mobile app [47] managed to break existing habits and promote more conscious and reflective decisions in participants when purchasing food. Similarly, a quantitative mobile app study [46] showed that a simplified eco-score improved sustainable purchase decisions (*p* < 0.001), with eco-score credibility identified as a strong driver of perceived value (i.e., users’ subjective evaluation of the usefulness and relevance of the digital tool; b = 0.31, *p* < 0.001).

Ultimately, *The Green Eating Project* [51] (Web-Based Platforms Section) found that participants using the web-platform were significantly more encouraged to reflect on and make more informed and deliberate decisions about food compared to controls (*p* < 0.001).

##### Nudging

Nudging was used as a psychological strategy in different ways across 31% (*n* = 5/16) of the selected studies [37,43,45,52,54].

N = 4 (25%) studies applied nudging in VR environments [43,45,52,54]. One study using VR (Virtual Reality Section [43]) applied impactful messages at the point of purchase in a virtual supermarket to steer participants toward eco-friendly choices with an increase in this variable compared to controls (*p* < 0.001). Visual contrast (e.g., red tables reducing red meat selection, *p* = 0.038) was instead applied in another VR study [52] to influence food choices in virtual environments. Another VR-based intervention [45] employed normative feedback (i.e., information on how their behavior compared to that of others) to motivate behavior change. Finally, VR images of the consequences of plastic on animals managed to impact choice of meal (*p* = 0.04) but not meal packaging selection [54].

Gamified nudges were also used in a mobile app to award points (“kudos”) for engaging in sustainable actions (including healthy and sustainable food choices). The app managed to reduce food waste in this qualitative study [37].

##### Self-Efficacy

Self-efficacy was addressed in 31% (*n* = 5/16) of the included studies [43,44,45,51,53], often as an outcome measure to assess participants’ confidence in engaging in healthy and sustainable eating behaviors.

This strategy was implemented in *n* = 3 (19%) VR studies (Virtual Reality Section). One study [43] provided messages to stimulate personal response efficacy beliefs (i.e., the belief that one is able to contribute to the solution of a problem), which predicted an increase in sustainable food choices (β = 0.30, *p* = 0.002). Personal response efficacy beliefs also maintained behavior change at FU (1–2 weeks). Plechatá et al. (2022) using VR also found that self-efficacy increased, showing the impact of food choices on a simulated environment significantly predicted intentions to increase healthy and sustainable eating behaviors (b = 0.35, 95% CI [0.13, 0.58], *p* = 0.003 [44]). The authors [45] also found by using the same VR software that normative feedback increasing self-efficacy reduced dietary footprint more than the control condition (d = 0.4), with effects persisting at FU (1 week).

N = 2 studies (13%), instead, implemented this strategy on web platforms (Web-Based Platforms Section) [51,53]. More specifically, participants of the qualitative study about “*The Green Hub*” [53] reported increased self-efficacy in replacing meat with plant-based proteins. “The Green Eating Project” [51], instead, found significant improvements in self-efficacy when adopting healthy and sustainable food choices at school (η^2^ = 0.03; *p* < 0.001), but not at home (η^2^ = 0.006).

##### Self-Monitoring

Self-monitoring was implemented in 25% (*n* = 3/16) of the studies [36,42,47], specifically on mobile apps (Web-Based Platforms Section) or instant messaging (Instant Messaging Systems (WhatsApp) Section).

In the qualitative study by Flaherty et al. (2020) [47] mentioned above (Mobile Apps Section), participants using the mobile app identified personal monitoring as one of the most effective techniques for behavior change. Similarly, another study [42] used in-app reminders to monitor participant’s weekly consumption of meat and legumes, which had promising effects on meat reduction, as mentioned in previous sections.

One study used WhatsApp messages [36] to encourage participants to fill in an online dietary diary to monitor their nutritional habits, which promoted motivation (e.g., intentions to eat ≤1 servings of processed meat in the following week, *p* < 0.008) and reduced meat consumption (*p* < 0.001) compared to controls.

##### Emotion Regulation

Emotion regulation was employed in 19% (*n* = 3/16) of the studies [36,41,48] to promote healthy and sustainable eating behaviors.

Two studies (*n* = 2, 13%) used mobile apps to influence emotional process and promote healthy and sustainable eating behaviors. Carfora et al. (2024) [41] (see Mobile Apps Section) reported that tailoring messages to users’ motivational focus and emotions (e.g., pride, guilt) significantly improved attitudes toward the app (F(1, 393) = 6.53, *p* = 0.011), desire (F = 7.36, *p* = 0.007), and intention (F = 4.64, *p* = 0.032) to use it to improve their eating habits. Weber (2021) [46] demonstrated that eco-score rankings increased sustainable food choices (*p* < 0.001) by reducing decision uncertainty and fostering positive emotional states (e.g., confidence, assurance) during food selection, which functioned as an indirect form of emotion regulation.

Another intervention [36] employed anticipated regret, a TPB-based strategy, to reduce red meat consumption by encouraging participants to reflect on future emotional consequences through WhatsApp messages. Using this strategy, participants were motivated to reduce their weekly meat intake, with the intervention group consuming significantly fewer portions than controls (1.74 vs. 3.29, *p* < 0.001).

## 4. Discussion

This systematic narrative review examines how digital technologies can support the promotion of healthy and sustainable eating behaviors in the general population, with a particular focus on categorizing the different psychological factors and behavioral strategies implemented in the available digital interventions to promote these food choices. A total of 16 studies were included, focusing on technological interventions designed to foster food choices that benefit both individual health and environmental sustainability. A major challenge in synthesizing the literature was the lack of a consistent set of term associated with “sustainable diets”, despite existing guidelines such as those from the FAO [15], which highlight the importance of nutritional adequacy alongside ecological responsibility. Many studies focused on isolated aspects, such as reducing meat consumption [55] or limiting food waste [56], without addressing the multidimensional nature of healthy and sustainable eating.

Due to the novelty of the field, broad inclusion criteria were adopted, allowing for heterogeneity in study design, sample size, and setting. Although heterogeneity led to the impossibility to perform a quantitative synthesis of results (e.g., through a meta-analytic approach), the selected studies provided promising results on the role of various digital tools implementing diverse psychological and behavioral strategies to increase healthy and sustainable dietary practices.

Regarding methodologies, the majority of the studies included in this review adopted an RCT design [35,36,41,42,43,44,45,46], which remains relatively underused in the broader literature on digital interventions for other sustainability-related behaviors [57,58]. A smaller but still relevant proportion of the selected articles were non-controlled studies [37,47,48,49,50,51,52,53], limiting the possibility of attributing observed effects specifically to the digital intervention and reducing causal inference regarding intervention effects. Although RCTs allow for greater control and reduced bias, only a few studies included FUs, posing a limitation regarding the durability of behavior change. This absence may reflect the recent emergence of this research field, with most studies published in the last four years. Furthermore, even when FUs were conducted (e.g., 8–11 weeks [47]), short duration of FUs and small sample sizes remained a critical issue. Compared to systemic, community-based interventions that promote deeper cultural shifts [59], digital interventions promoting healthy and sustainable eating habits tend to focus on short-term, individual-level behavioral prompts [60,61], raising questions about their long-term transformative potential.

The first aim of the systematic narrative review was to evaluate the effects of digital tools in promoting healthy and sustainable eating behaviors. The interventions with mobile apps and VR, which represented the most employed platforms, consistently showed positive impacts on targeted behaviors, such as reductions in red and processed meat consumption [36,42,52,53], increased selection of plant-based options [42,45,54], and improvements in food-related awareness [37,44,45,51,53] and decision-making [43,46,50].

It is important to highlight that other technologies, such as web-based platforms and instant messaging systems, also offered some promising findings but remain underused in this field to date. For instance, web-based interventions [51,53] and a WhatsApp-based intervention [36] successfully encouraged healthier eating behaviors. However, despite the encouraging nature of these findings, more rigorous, large-scale studies and RCTs with longer FUs are needed to further explore the potential of these underused digital tools in promoting healthy and sustainable eating behaviors.

The second aim of this systematic narrative review was to explore and systematically categorize which psychological factors and behavioral strategies have been implemented by the found digital tools to promote healthy and sustainable eating behaviors.

Awareness emerged as the most consistently targeted psychological factor, present in the majority of the included studies [35,37,42,44,45,50,51,53,54]. It was typically used to increase participants’ knowledge of sustainable food practices and environmental impact. In several studies, awareness was enhanced through educational content [42,51,53], gamified learning [37], or interactive experiences [44,45]. In some cases, increased awareness was directly linked to more sustainable food choices, as seen in interventions using infographics [35,54] or personalized feedback [50]. Environmental awareness, indeed, frequently emerged in the literature as a fundamental strategy to promote sustainable behaviors, even outside of food choices [58,62,63].

Decision-making and emotion regulation were also addressed in some of the selected studies [36,41,43,46,47,50,51]. Decision-making was often supported through strategies like eco-scores on food labels [46], informative feedback [43,50,51], motivational messages [41] or narrative elements promoting cognitive engagement [47]. Emotion regulation was mostly operationalized through anticipated emotions (such as regret, pride, or guilt) used to influence dietary intentions [36,41]. These studies reinforces the body of literature [64,65] highlighting that emotion regulation strategies can increase participants’ motivation and willingness to engage in healthy and sustainable food choices, especially when messages are tailored to the users’ values or identity [46]. However, it is surprising to note that only two studies (and no VR study) implemented this strategy, which has been successfully used in other clinical and non-clinical fields for the reduction of dysfunctional eating behaviors through technology (especially VR [32,66]) or the promotion of other sustainable habits [64].

Self-monitoring was implemented in few of the selected studies [36,42,47], and exclusively through mobile apps and instant messaging. In these studies, participants were encouraged to track their eating habits, set personal goals, or receive feedback on their progress. These functions were integrated into broader persuasive systems, often supported by reminders or motivational prompts. Self-efficacy and response efficacy were targeted particularly through immersive environments and feedback in VR studies [43,44,45,51,53] and informative content on web platforms [51,53]. These studies showed that when users could visualize the environmental or health impact of their choices in real-time, their confidence in making sustainable decisions increased accordingly [62,67]. This is in line with the literature showing that increasing self-efficacy and individuals’ belief that they can contribute to change can encourage them toward pro-environmental behaviors [62,67].

The findings of this review are consistent with previous research demonstrating that digital technologies, particularly mobile apps and immersive environments such as VR, can positively influence eating behaviors when they incorporate psychological strategies such as emotional engagement, real-time feedback, and guided decision-making [30,32,34]. Moreover, the incorporation of Design for Sustainable Behavior (DfSB) principles [26] within mobile applications and digital interfaces reflects a growing design philosophy in which feedback mechanisms, visual cues, and interface structure are intentionally developed to guide users toward more ecologically sound behavior. These principles align with evidence from ecological and environmental psychology showing that seemingly small design adjustments (e.g., food labeling systems or color-based nudges) can influence consumer choices at both cognitive and emotional levels [23,46,52].

### Limitations and Future Research Directions

The promising results of the current systematic narrative review must be interpreted in light of several methodological limitations. First of all, the psychological and behavioral strategies were not always explicitly reported in the included studies, and in several cases they had to be inferred by the authors during the review process, which may introduce a degree of subjectivity in their categorization and the way results of the systematic review were summarized. Similarly, a further limitation concerns the selection of search keywords, which may not have captured all relevant studies due to the lack of a standardized list of terms for healthy and sustainable eating behaviors in the literature. Future research should therefore aim to adopt more theory-driven and explicitly articulated intervention frameworks, as well as greater transparency in reporting the psychological and behavioral strategies implemented in digital tools, to reduce ambiguity and facilitate more consistent synthesis across studies.

Moreover, methodological issues emerged also in relation to the selected studies, which rarely achieved a strong quality score. Indeed, a substantial proportion of studies relied on small samples or short-term designs with no or very short FUs. However, some differences emerged across technologies: mobile app-based interventions more frequently included larger samples and FUs compared to studies employing VR. Future research should therefore prioritize study designs that combine the scalability of mobile app-based interventions with more rigorous methodological features and longer FU (e.g., longer than 3 months) and to also extending these standards to VR studies to better assess their long-term effects and real-world applicability.

Finally, a further limitation of this review is the inability to conduct a quantitative synthesis through meta-analytic methods, due to the substantial heterogeneity of the included studies in terms of digital technologies, targeted behaviors, outcome measures (which in several cases were not standardized questionnaires, but rather ad hoc or qualitative instruments), and study designs. Future studies should therefore aim to adopt more standardized outcome measures and harmonized study designs, which would enable quantitative syntheses and meta-analytic comparisons across digital technologies and strategies. As meta-analytic approaches represent the most robust method for estimating overall intervention effectiveness, the absence of a meta-analysis in the present review limits the possibility of drawing definitive conclusions regarding the magnitude of effects and of comparing the effectiveness of different technologies or strategies.

## 5. Conclusions

This systematic narrative review tried to summarize the growing, but still preliminary findings in the literature showing the potential benefits of using digital technologies, particularly mobile applications and VR, in promoting healthier and more sustainable eating behaviors in the general population. Although characterized by several methodological limitations, the presented studies also suggest that integrating psychological strategies into these digital tools, such as awareness, emotion regulation, decision-making, and self-efficacy, could promote eating behavior change towards healthier and more sustainable choices. Theoretically, this can reinforce the centrality of cognitive-affective factors in shaping sustainable consumption [23,27] and encourages the integration of design-based frameworks such as Design for Sustainable Behavior [26] within psychological intervention models.

From a clinical and psychoeducational perspective, results from the current systematic narrative review suggest that digital tools could be used complementarily with more traditional health promotion programs by offering scalable, engaging, and user-centered pathways to behavior change. This aligns with previous findings on the use of technology-enhanced interventions in both educational [33,34] and clinical contexts [30,32]. However, additional interdisciplinary collaboration among psychologists, designers, and sustainability experts is essential to develop digital interventions that are both clinically effective and environmentally meaningful. Moreover, longitudinal and real-world studies are needed to evaluate the sustained impact of these tools on both individual behavior and broader systems of food consumption.

## Figures and Tables

**Table 1:** Population, intervention, comparison, outcomes and study (PICOS) table for inclusion and exclusion criteria.

PICOS	Inclusion Criteria	Exclusion Criteria
**Population**	Adults, adolescents, children, or mixed populations Male, female, or mixed gender Any sample size	Individuals with medical conditions Absence of participants
**Intervention**	Use of technologies to promote sustainable dietary behaviors	Use of technologies targeting other sustainability-related behaviors (e.g., waste reduction, reuse, recycling, use of public transport, tree planting, etc.) Questionnaire validation studies Proof of concept (POC) studies
**Comparison group**	Studies with or without a control group	-
**Outcome**	Changes in eating behavior Reduction in the ecological impact of food consumption Improvements in personal and environmental health	Other outcome variables
**Study design**	Randomized controlled trials (RCTs) Longitudinal studies Retrospective studies Cross-sectional studies Any research setting	Reviews Meta-analyses Case studies Commentaries or conference proceedings Theses Book chapters Pilot studies

## Data Availability

This is a systematic narrative review with no quantitative synthesis. Data can therefore be found in the original version of the selected articles.

## Supplementary Materials

The following supporting information can be downloaded at https://www.mdpi.com/article/10.3390/nu18040645/s1, Table S1: Characteristics and results of the included studies; Table S2: Criteria and scores used for risk of bias assessment and evaluation of the quality of the included studies; Table S3: Risk of bias assessment and evaluation of the quality of the included studies; Table S4: PRISMA checklist [68].

## Abbreviations

The following abbreviations are used in this manuscript:

AI	Artificial Intelligence
AR	Augmented Reality
BMI	Body Mass Index
DfSB	Design for Sustainable Behavior
EDs	Eating Disorders
FAO	Food and Agriculture Organization
FU	Follow-Up
M	Mean
NIH	National Institutes of Health (nel testo: National Institutes of Mental Health, strumento NIH)
PICOS	Population, Intervention, Comparison, Outcome, Study Design
POC	Proof of Concept
PRISMA	Preferred Reporting Items for Systematic Reviews and Meta-Analyses
PROSPERO	International Prospective Register of Systematic Reviews
RCT	Randomized Controlled Trial
SD	Standard Deviation
SEM	Structural Equation Modeling
TPB	Theory of Planned Behavior
VR	Virtual Reality
WHO	World Health Organization

## References

[1] World Health Organization One Health. https://www.who.int/news-room/questions-and-answers/item/one-health.

[2] Willett W., Rockström J., Loken B., Springmann M., Lang T., Vermeulen S., Garnett T., Tilman D., DeClerck F., Wood A. (2019). Food in the Anthropocene: The EAT–Lancet Commission on Healthy Diets from Sustainable Food Systems. Lancet.

[3] Poore J., Nemecek T. (2018). Reducing Food’s Environmental Impacts through Producers and Consumers. Science.

[4] O’Neil A., Quirk S.E., Housden S., Brennan S.L., Williams L.J., Pasco J.A., Berk M., Jacka F.N. (2014). Relationship Between Diet and Mental Health in Children and Adolescents: A Systematic Review. Am. J. Public Health.

[5] Jacka F.N., Kremer P.J., Berk M., de Silva-Sanigorski A.M., Moodie M., Leslie E.R., Pasco J.A., Swinburn B.A. (2011). A Prospective Study of Diet Quality and Mental Health in Adolescents. PLoS ONE.

[6] Riebel L. (2001). Consuming the Earth: Eating Disorders and Ecopsychology. J. Humanist. Psychol..

[7] Bremner J.D., Moazzami K., Wittbrodt M.T., Nye J.A., Lima B.B., Gillespie C.F., Rapaport M.H., Pearce B.D., Shah A.J., Vaccarino V. (2020). Diet, Stress and Mental Health. Nutrients.

[8] Angelos J.A., Arens A.L., Johnson H.A., Cadriel J.L., Osburn B.I. (2017). One Health in Food Safety and Security Education: Subject Matter Outline for a Curricular Framework. One Health.

[9] Paris J.M.G., Escobar N., Falkenberg T., Gupta S., Heinzel C., Junior E.V., Jolliet O., Borgemeister C., Nöthlings U. (2024). Optimised Diets for Achieving One Health: A Pilot Study in the Rhine-Ruhr Metropolis in Germany. Environ. Impact Assess. Rev..

[10] Żakowska-Biemans S., Pieniak Z., Kostyra E., Gutkowska K. (2019). Searching for a Measure Integrating Sustainable and Healthy Eating Behaviors. Nutrients.

[11] Brytek-Matera A. (2021). Vegetarian Diet and Orthorexia Nervosa: A Review of the Literature. Eat. Weight Disord.-Stud. Anorex. Bulim. Obes..

[12] Devecchi A., Ponzo V., Favaro E., Goitre I., Stella B., Pagliuca G., Cuniberti F., Abbate-Daga G., Donini L.M., Bo S. (2025). Eco-Concerns and Risk for Eating Disorders and Orthorexia Nervosa. J. Affect. Disord..

[13] Tecuta L., Gardini V., Tomba E. (2025). Climate Change Worry and Eating-Related Eco-Concern: A Network Analysis of Psychological and Behavioral Correlates in the General Population. J. Eat. Disord..

[14] Szulc P., Willich K., Gogga P. (2025). Association Between Orthorexia and Plant-Based Diets—Is There a Vicious Cycle?. Nutrients.

[15] Food and Agriculture Organization (2012). Sustainable Diets and Biodiversity: Directions and Solutions for Policy, Research and Action.

[16] Graça J., Calheiros M.M., Oliveira A. (2015). Attached to Meat? (Un)Willingness and Intentions to Adopt a More Plant-Based Diet. Appetite.

[17] Michel F., Hartmann C., Siegrist M. (2021). Consumers’ Associations, Perceptions and Acceptance of Meat and Plant-Based Meat Alternatives. Food Qual. Prefer..

[18] Lourenco C.E., Nunes-Galbes N.M., Borgheresi R., Cezarino L.O., Martins F.P., Liboni L.B. (2022). Psychological Barriers to Sustainable Dietary Patterns: Findings from Meat Intake Behaviour. Sustainability.

[19] Graça J., Godinho C.A., Truninger M. (2019). Reducing Meat Consumption and Following Plant-Based Diets: Current Evidence and Future Directions to Inform Integrated Transitions. Trends Food Sci. Technol..

[20] Kollmuss A., Agyeman J. (2002). Mind the Gap: Why Do People Act Environmentally and What Are the Barriers to pro-Environmental Behavior?. Environ. Educ. Res..

[21] Lea E.J., Crawford D., Worsley A. (2006). Public Views of the Benefits and Barriers to the Consumption of a Plant-Based Diet. Eur. J. Clin. Nutr..

[22] González-Azcárate M., Cruz Maceín J.L., Bardají I. (2021). Why Buying Directly from Producers Is a Valuable Choice? Expanding the Scope of Short Food Supply Chains in Spain. Sustain. Prod. Consum..

[23] Sörqvist P. (2016). Grand Challenges in Environmental Psychology. Front. Psychol..

[24] Ebinger F., Buttke L., Kreimeier J. (2022). Augmented and Virtual Reality Technologies in Education for Sustainable Development: An Expert-Based Technology Assessment. TATuP-Z. Tech. Theor. Prax..

[25] Isensee C., Teuteberg F., Griese K.M. (2022). Exploring the Use of Mobile Apps for Fostering Sustainability-Oriented Corporate Culture: A Qualitative Analysis. Sustainability.

[26] Chiu M.-C., Kuo T.-C., Liao H.-T. (2020). Design for Sustainable Behavior Strategies: Impact of Persuasive Technology on Energy Usage. J. Clean. Prod..

[27] Ajzen I. (1991). The Theory of Planned Behavior. Organ. Behav. Hum. Decis. Process..

[28] Linardon J., Cuijpers P., Carlbring P., Messer M., Fuller-Tyszkiewicz M. (2019). The Efficacy of App-Supported Smartphone Interventions for Mental Health Problems: A Meta-Analysis of Randomized Controlled Trials. World Psychiatry.

[29] Ciążyńska J., Maciaszek J. (2022). Various Types of Virtual Reality-Based Therapy for Eating Disorders: A Systematic Review. J. Clin. Med..

[30] Linardon J., Shatte A., Rosato J., Fuller-Tyszkiewicz M. (2022). Efficacy of a Transdiagnostic Cognitive-Behavioral Intervention for Eating Disorder Psychopathology Delivered through a Smartphone App: A Randomized Controlled Trial. Psychol. Med..

[31] Porras-Garcia B., Ferrer-Garcia M., Serrano-Troncoso E., Carulla-Roig M., Soto-Usera P., Miquel-Nabau H., Fernández-Del castillo Olivares L., Marnet-Fiol R., de la Montaña Santos-Carrasco I., Borszewski B. (2021). AN-VR-BE. A Randomized Controlled Trial for Reducing Fear of Gaining Weight and Other Eating Disorder Symptoms in Anorexia Nervosa through Virtual Reality-Based Body Exposure. J. Clin. Med..

[32] Gardini V., Grandi S., Tomba E. (2025). A Novel Transdiagnostic Approach to the Prevention of Eating Disorders Using Virtual Reality: Preliminary Evaluation of the H.O.M.E. Intervention. Clin. Psychol. Psychother..

[33] Leme A.C.B., Philippi S.T., Thompson D., Nicklas T., Baranowski T. (2019). “Healthy Habits, Healthy Girls—Brazil”: An Obesity Prevention Program with Added Focus on Eating Disorders. Eat. Weight Disord.-Stud. Anorex. Bulim. Obes..

[34] Pulimeno M., Piscitelli P., Pacella G., Rizzo E., Galante B., Colazzo S. (2019). Health Pedagogy and Narrative-Based Strategies to Promote Healthy Eating Behaviours and Prevent Obesity in Schoolchildren: An Experimental Protocol Designed in Salento. JDREAM J. Interdiscip. Res. Appl. Med..

[35] Herrewijn L., De Groeve B., Cauberghe V., Hudders L. (2021). VR Outreach and Meat Reduction Advocacy: The Role of Presence, Empathic Concern and Speciesism in Predicting Meat Reduction Intentions. Appetite.

[36] Carfora V., Caso D., Conner M. (2017). Randomised Controlled Trial of a Text Messaging Intervention for Reducing Processed Meat Consumption: The Mediating Roles of Anticipated Regret and Intention. Appetite.

[37] Haas R., Aşan H., Doğan O., Michalek C.R., Karaca Akkan Ö., Bulut Z.A. (2022). Designing and Implementing the MySusCof App—A Mobile App to Support Food Waste Reduction. Foods.

[38] Moher D., Liberati A., Tetzlaff J., Altman D.G., PRISMA Group (2009). Preferred Reporting Items for Systematic Reviews and Meta-Analyses: The PRISMA Statement. PLoS Med..

[39] Methley A.M., Campbell S., Chew-Graham C., McNally R., Cheraghi-Sohi S. (2014). PICO, PICOS and SPIDER: A Comparison Study of Specificity and Sensitivity in Three Search Tools for Qualitative Systematic Reviews. BMC Health Serv. Res..

[40] National Institutes of Mental Health (2021). Study Quality Assessment Tools. https://www.nhlbi.nih.gov/health-topics/study-quality-assessment-tools.

[41] Carfora V., Festa S., Pompili S., Azzena I., Guidetti M., Scaglioni G., Carraro L., Lenzi M., Scatolon A., Cavazza N. (2024). Regulatory Fit to Enhance User Engagement with an App Promoting Healthy and Sustainable Eating. An Experimental Study to Match Regulatory Concern and Anticipated Emotions. Sustainability.

[42] Carfora V., Catellani P. (2023). Legumes or Meat? The Effectiveness of Recommendation Messages towards a Plant-Based Diet Depends on People’s Identification with Flexitarians. Nutrients.

[43] Meijers M.H.C., Smit E.S., de Wildt K., Karvonen S.-G., van der Plas D., van der Laan L.N. (2022). Stimulating Sustainable Food Choices Using Virtual Reality: Taking an Environmental vs Health Communication Perspective on Enhancing Response Efficacy Beliefs. Environ. Commun..

[44] Plechatá A., Morton T., Perez-Cueto F., Makransky G. (2022). Why Just Experience the Future When You Can Change It: Virtual Reality Can Increase Pro-Environmental Food Choices through Self-Efficacy. Technol. Mind Behav..

[45] Plechatá A., Morton T., Perez-Cueto F.J.A., Makransky G. (2022). A Randomized Trial Testing the Effectiveness of Virtual Reality as a Tool for Pro-Environmental Dietary Change. Sci. Rep..

[46] Weber A. (2021). Mobile Apps as a Sustainable Shopping Guide: The Effect of Eco-Score Rankings on Sustainable Food Choice. Appetite.

[47] Flaherty S.J., McCarthy M.B., Collins A.M., McCafferty C., McAuliffe F.M. (2020). A Phenomenological Exploration of Change towards Healthier Food Purchasing Behaviour in Women from a Lower Socioeconomic Background Using a Health App. Appetite.

[48] Agustina R., Febriyanti E., Putri M., Martineta M., Hardiany N.S., Mustikawati D.E., Hanifa H., Shankar A.H. (2022). Development and Preliminary Validity of an Indonesian Mobile Application for a Balanced and Sustainable Diet for Obesity Management. BMC Public Health.

[49] Azzena I., Pompili S., Festa S., Lenzi M., Carraro L., Guidetti M., Cavazza N., Catellani P., Carfora V. (2025). Psychosocial Predictors of Downloading a Mobile App Promoting Healthy and Sustainable Eating. Cogent Psychol..

[50] De Croon R., Segovia-Lizano D., Finglas P., Abeele V.V., Verbert K. (2025). An Explanation Interface for Healthy Food Recommendations in a Real-Life Workplace Deployment: User-Centered Design Study. JMIR mHealth uHealth.

[51] Monroe J.T., Lofgren I.E., Sartini B.L., Greene G.W. (2015). The Green Eating Project: Web-Based Intervention to Promote Environmentally Conscious Eating Behaviours in US University Students. Public Health Nutr..

[52] Wan X., Qiu L., Wang C. (2022). A Virtual Reality-Based Study of Color Contrast to Encourage More Sustainable Food Choices. Appl. Psychol. Health Well-Being.

[53] Ghammachi N., Mihrshahi S., Ronto R. (2022). Web-Based Experiential Nutrition Education Intervention “The Green Hub” to Promote Sustainable and Healthy Diets among Young Adults in Australia. Sustainability.

[54] Farias A.R., Lane H., Killingsworth J., Warden J.M., Wais S. (2024). Exploring the Interplay of Pro-Environmental Attitudes, Dietary Choices, and Packaging Preferences: A Virtual Reality Restaurant Scenario Study. Challenges.

[55] Springmann M., Wiebe K., Mason-D’Croz D., Sulser T.B., Rayner M., Scarborough P. (2018). Health and Nutritional Aspects of Sustainable Diet Strategies and Their Association with Environmental Impacts: A Global Modelling Analysis with Country-Level Detail. Lancet Planet. Health.

[56] Meyer N., Reguant-Closa A. (2017). “Eat as If You Could Save the Planet and Win!” Sustainability Integration into Nutrition for Exercise and Sport. Nutrients.

[57] Larochelle C., Alwang J., Travis E., Barrera V.H., Dominguez Andrade J.M. (2019). Did You Really Get the Message? Using Text Reminders to Stimulate Adoption of Agricultural Technologies. J. Dev. Stud..

[58] Turan Çimşir B., Uzunboylu H. (2019). Awareness Training for Sustainable Development: Development, Implementation and Evaluation of a Mobile Application. Sustainability.

[59] Goh L.M.L., Wong A.X.Y., Ang G.Y., Tan A.S.L. (2017). Effectiveness of Nutrition Education Accompanied by Cooking Demonstration. Br. Food J..

[60] Barbour L., Lindberg R., Woods J., Charlton K., Brimblecombe J. (2022). Local Urban Government Policies to Facilitate Healthy and Environmentally Sustainable Diet-Related Practices: A Scoping Review. Public Health Nutr..

[61] Drewnowski A., Monterrosa E.C., de Pee S., Frongillo E.A., Vandevijvere S. (2020). Shaping Physical, Economic, and Policy Components of the Food Environment to Create Sustainable Healthy Diets. Food Nutr. Bull..

[62] Plohl N., Bakračevič K., Musil B., Rutar M., Tement S., Horvat M. (2026). Subjective Norms and Pro-Environmental Behavior: The Role of Environmental Awareness and Self-Efficacy. Curr. Res. Ecol. Soc. Psychol..

[63] Godinho Filho M., Gonella J.d.S.L., Latan H., Ganga G.M.D. (2024). Awareness as a Catalyst for Sustainable Behaviors: A Theoretical Exploration of Planned Behavior and Value-Belief-Norms in the Circular Economy. J. Environ. Manag..

[64] Abbas M., Iftikhar H. (2025). Face Your Feelings, Save the Planet: How We Regulate Emotions Affects pro-Environmental Behavior. J. Environ. Psychol..

[65] Brosch T., Steg L. (2021). Leveraging Emotion for Sustainable Action. One Earth.

[66] Dol A., van Gemert-Pijnen L., Schwartz L.M., Velthuijsen H., Bode C. (2023). Exploring Tailored Virtual Emotion Regulation Approaches for Individuals with Emotional Eating. J. Eat. Disord..

[67] Zhang J., Cao A. (2025). The Psychological Mechanisms of Education for Sustainable Development: Environmental Attitudes, Self-Efficacy, and Social Norms as Mediators of Pro-Environmental Behavior Among University Students. Sustainability.

[68] Page M.J., McKenzie J.E., Bossuyt P.M., Boutron I., Hoffmann T.C., Mulrow C.D., Shamseer L., Tetzlaff J.M., Akl E.A., Brennan S.E. (2021). The PRISMA 2020 statement: An updated guideline for reporting systematic reviews. BMJ.

