# Supplementary material for: Digital Tools for the Promotion of Healthy and Sustainable Eating Behaviors in the General Population: A Systematic Review of the Literature
Source: Nutrients. 2026 Feb 16;18(4):645. doi: 10.3390/nu18040645 (PMC12943175; doi:10.3390/nu18040645)
Supplement: Supplementary file 1 [file nutrients-18-00645-s001.zip › nutrients-4123560-supplementary.pdf]

Supplementary Table S1. Characteristics and results of the included studies

Article	Sample size (%F - %M)<br>Median±SD Age (Age Range)	Sample type	Study design<br><br>(+ Follow-up when present)	Type of technology used	Software Name	Assessment tools	Psychological and behavioral strategies employed<br>(+ reference theories when present)	Main results of the study
Agustina et al. (2022)	G1. 39 (23%- 77%)<br>G2. 53 (64%-36%)<br>G3. 30 (100%-0%)<br><br>M=N/A (range 19 - 64 years)	G1. GP (government employees)<br>G2. GP (healthy adults)<br>G3. Women with obesity or overweight (BMI ≥ 25.0 kg/m^2)	Multi-phase study	Mobile App	EatsUp®	Mobile app for food tracking<br>Ad hoc interview<br>Concordance test (Bland-Altman plot)	Self-monitoring<br><br>Motivation<br><br>Emotion regulation	The EatsUp® app can support the monitoring of a balanced and sustainable dietary practice, providing nutritional data and being comparable with conventional (paper-based) dietary assessment tools (p>0.05, thus within the confidence interval for agreement)...<br>EatsUp® was positively evaluated in terms of content, graphics and flow of the app. 70% of respondents rated 14 out of 17 parameters as "good".<br>Participants evaluated the app as easy, simple, attractive, and informative in monitoring calories, dietary composition and providing nutritional facts.
Carfora et al. (2017)	N=112 (56%-44%)<br><br>M=19.37 ± 1.55	GP college students (non-vegetarian/vegan)	Randomized Control Trial<br><br>(FU= 1 week)	Text messages on WhatsApp	N/A	Online food diary<br><br>Ad hoc TPB questionnaire	Self-monitoring<br><br>Emotion regulation (in particular, anticipation of regret)	The intervention was effective in promoting both intentions and the actual reduction of meat consumption.<br>Participants in the experimental condition ate an average of 1.74 servings of meat per week, a significantly smaller amount than the control group (M=3.29, p<.001).

								At the post-intervention, the experimental group reported significantly more intentions to eat ≤1 servings of processed meat in the following week, compared to the control group ($p<.008$).
Carfora & Catellani (2023)	T1: 428 (48%-52%) M=34.73 ± 6.8<br><br>T2: 296 (47%-53%) M=35.29 ± 16.44<br><br>T3: 221 (53%-47%) M=32.25 ± 16.32<br><br>18 – 80 years old	GP healthy adults	Randomized Control Trial<br><br>(FU = 2 weeks and 1 month)	Mobile App	PsyMe	Mobile App<br><br>Ad hoc online questionnaires (via the app)	Self-monitoring<br><br>Awareness	The results show that, in the short term, messages of addition (rather than meat replacement) increase the consumption of legumes, especially in those who evaluate flexitarians negatively. In the long term, the increase in legume consumption is only recorded when the addition messages are combined with dynamic norms (e.g. info that more and more people are implementing that behavior). In addition, there was a significant interaction effect between time spent and messages with regard to both legume and meat consumption ($p<.05$).

Carfora et al. (2024)	N=401<br>(66.3% - 32.2%) + 1.5% not specified<br><br>M=35.57 ± 14.52<br><br>18-80 years old	GP college students and healthy adults	Randomized Control Trial	Messages to encourage the use of mobile apps for sustainable food	N/A	Ad hoc questionnaires with validated questionnaire items	Emotion regulation (in particular anticipated emotion, regulatoru fit)<br><br>Decision making<br><br>(Reference theories:<br>RFT; TPB)	Regulatory messages in line with the user's motivational focus (regulatory fit) have significantly increased:<br>• the attitude towards the app ($F(1,393)=6.53$, $p=.011$);<br>• desire ($F=7.36$, $p=.007$);<br>• intention ($F=4.64$, $p=.032$);<br>• download rate ($\chi^2=5.21$, $p=.022$).<br>Therefore, people who received a message in line with their motivational orientation (e.g. growing up with positive emotions) were more likely to have a positive attitude and download the app.

De Croon et al. (2025)	N (phase 3) = 136 (65% - 35%)<br><br>M=41 ± 10<br><br>23 - 61 years	GP corporate employees	Uncontrolled study (iterative process)	Mobile App	N/A	FFQ<br>ResQue Questionnaire<br><br>In-app feedback<br><br>Quality methods	Awareness<br><br>Self-regulation<br><br>Decision making	Only 41 of 136 participants (30%) completed the entire study and post-intervention questionnaires.<br><br>ResQue (N=41): 33/41 found explanations clear<br><br>Only 22/41 felt that the recommendations reflected their preferences<br><br>27/41 expressed confidence in the system<br><br>Dietary changes observed included increased intake from vegetables and fruits compared to carbohydrates from starchy sources, reduced consumption of snacks and sweets in some participants, decreased reliance on dairy products and increased contribution of vegetables in total protein for some
Farias et al. (2023)	N= 22 (73%-27%)<br><br>M=23.4 ± 8.9	GP students	Randomized Control Trial	VR	N/A	PS<br>PER<br>GPI	Awareness<br><br>Nudging	The studio used a VR scenario with a virtual waiter offering vegetarian and non-vegetarian dishes, and recyclable and non-recyclable packaging, with a warning message of an animal hurting from plastic exposure.<br>Participants' were assessed on established scales to measure sustainability awareness (i.e. PS, PER and GPI).<br>Those who choose vegetarian meals scored higher on these scales (M=6.4) than those who eat meat/fish (M=5.7), especially women (M=0.4), as well as those who choose recyclable packaging materials (M=6.1) compared to those

								who choose non-recyclable packaging materials (M=5.3). Fisher's test (p=.04) showed that the VR message had a significant influence on choice of meal, but no influence on packaging selection.
Flaherty et al. (2020)	N=10 (100%-0%)<br><br>M=N/A (range 30-45 years)	GP adult women	Phenomenological qualitative study<br><br>(FU= 8-11 weeks)	Mobile App	N/A	Mobile App<br>Ad hoc questionnaires<br>Semi-structured interviews<br>Remarks	Self-monitoring<br><br>Decision making	Considering questionnaire analyses and semi-structured interviews, conducted at baseline and in follow-up, the app seems to have facilitated the breaking of existing habits and promoted a more conscious and reflective approach to the decision to purchase food.<br>Personal monitoring, problem-solving, and behavioral stimuli have been identified as the most effective techniques for behavior change.
Ghammachi et al. (2022)	N=17 (87%-13%)<br><br>M=N/A (range 18-25 years)	GP healthy young adults	Pre-post study design	Web platform	The Green Hub	Green Eating Scale<br><br>Australian Health Survey<br><br>General Nutritrion Knowledge Questionnaire<br><br>FFQ	Self-efficacy<br><br>Awareness	Participants expressed positive feedback on the impact of "The Green Hub" program on their willingness to adopt sustainable and healthy diets. After the program, the consumption of red meat (from 40% to 26%), fast food (from 20% to 0%), and food waste (from 7% to 0%) has decreased, while the consumption of vegetable proteins (from 86.7% to 100%) and the purchase of 0 km (from 73.4% to 100%) and seasonal (from 83.3% to 100%) food have increased.

Haas et al. (2022)	N=121 (46%-54%)<br><br>M=26.4 (range 18-52 years)	GP adults and young adults	Uncontrolled study (iterative process)	Mobile App	MySusCof	uMARS	Awareness<br><br>Nudging	The results show that the app has a positive impact on awareness and intention to change behaviour around food waste, with 73% (n=88) of users indicating that they have become more aware of food waste through the use of the app.
Herrewijn et al. (2021)	N=84 (55%-45%)<br><br>M=29 ± 13.8	GP adults (Non-vegetarian/vegan)	Randomized Control Trial	VR<br>Video on slaughterhous e scandals	N/A	Ad hoc questionnaire	Awareness	VR increases the feeling of presence (p=.039), which can positively influence empathy (p<.001) and the intention to reduce meat consumption (p=.022). On the other hand, high values on the speciesism scale can reduce empathy (p<.001) and counteract the positive effects of VR in promoting changes in eating behavior.
Meijers et al. (2022)	N=249 (77%-23%)<br><br>M=21.56 ± 3.48	GP students	Randomized Control Trial<br><br>FU=1 and 2 weeks	VR	VirtuMart	Ad hoc questionnaire	Self-efficacy<br><br>Decision making<br><br>Nudging (impactful messages)	The results confirmed that the messages that pop up at the time of decision-making, led to more environmentally friendly food choices in the VR supermarket, $F(4, 241) = 16.80$ $p < .001$, $r2 = .20$, where the control condition differed significantly from all other conditions ($p < .001$). In addition, personal response efficacy beliefs were a larger predictor for product choices in the VR-supermarket ($\beta = .30$, $p = .002$).<br>Personal response efficacy beliefs also predicted pro-environmental food choices ($\beta=.30$, $p=.002$).

Monroe et al. (2015)	N=608 (77.8% - 22.2%)<br><br>M=18.9 ± 1.1	GP university students	Pre-post study design	Web platform	The Green Eating Project	Green Eating Survey	Self-efficacy<br><br>Awareness<br><br>Decision making	There was a significant increase in Green Eating Behaviour in the experimental group (from M=2.33±0.80 to M=2.60±0.81) compared to the control group (from M=2.45±0.81 to M=2.47±0.85) (F(1, 405df) = 13.89, p<.001, η2 = 0.03). A significant increase in Green Eating Knowledge was also found in the experimental group, compared to the control group (F(1, 407df) = 51.15, p <.001, η2 = 0.11). Significant improvements in "school-based" (η2=.03; p<.001) but not "home-based" self-efficacy (η2=.006) in adopting healthy and sustainable food choices.
Plechatá et al. (2022a)	N=123 (78%-22%)<br><br>M=25.03 ± 6.4	GP students	Randomized Control Trial<br><br>(FU= 1 week)	VR	N/A	Ad hoc questionnaire	Self-efficacy<br><br>Awareness<br><br>Nudging (regulatory feedback)	In this study, we investigated the effect of VR intervention on dietary footprint measured from 1 week before to 1 week after the intervention. The VR intervention significantly reduced the individual food footprint (d = 0.4, p = .034) compared to the control condition (who only completed the questionnaires, without the VR intervention). In addition, the VR condition increased response effectiveness (p=.045) and knowledge to a greater extent than control (p<.001).

Plechatá et al. (2022b)	N=90 (49%-40%-11% other)<br><br>M=14.3 ± 0.6	GP middle School Students	Randomized Control Trial	VR	N/A	Ad hoc questionnaires	Self-efficacy<br><br>Awareness	The condition mindfulness + self-efficacy led to a significant increase in pro-environmental intentions, b = 0.35, 95% CI [0.13, 0.58], p = .003, and knowledge transfer, b = 35.44, 95% CI [66.93, 3.95], t(87) = −2.24, p = .028, compared to awareness alone. Self-efficacy mediated these effects, while response efficacy did not have a significant impact on the outcome.
Wan et al. (2022)	N=52 (54%-46%)<br><br>M=20.85 ± 1.88	GP Young adults (non-vegetarian, normal weight)	Experimental within-subjects design	VR	Vizard 4.0	Ad hoc questionnaire	Nudging	The study manipulated the color of the virtual tables (red or green) and measured how this affected the participants' choices between meat or vegetable meals. The red table led to choosing less meat (61.2% vs 66.9%), and to a greater extent to choosing vegetarian dishes. Indeed, the comparisons revealed that meat dishes looked less attractive when presented on red tables than on green tables, t(51) = 2.42, p = 0.038, Cohen's d = 0.34, while vegetarian dishes looked comparably attractive when presented on red and green tables, t(51) = 1.69, p = 0.20.
Weber et al. (2021)	N=332 (54%-46%)<br><br>M=42.2	GP healthy adults	Randomized Control Trial	Mobile App	N/A	Ad hoc online questionnaire	Decision making<br><br>Awareness<br><br>Emotion regulation	The article showed that using eco ratings for food products can positively influence consumer choices towards more sustainable options (p<.001). The credibility of the eco-ranking information was identified as a strong driver of perceived value, b=0.31, p<.001.

Abbreviations. *%F: percentage of females in the sample; %M: percentage of males in the sample; BMI: Body Mass Index; CI: Confidence Interval; FFQ: Food Frequency Questionnaire; FU: follow-up G: group; GP: general population; GPI: Green Purchase Intention scale; M: mean; PER: Perceived Environmental Responsibility scale; PS: Perceived Seriousness of environmental behavior scale; RFT: Regulatory Focus Theory; TPB: Theory of Planned Behaviour; uMARS: user-friendly version of the Mobile Application Rating Scale; UTAUT: Unified Theory of Acceptance and Use of Technology; VR: virtual reality*

Supplementary Table S2. Criteria and scores used for risk of bias assessment and evaluation of the quality of the included studies

CRITERIA	STRONG	MODERATE	WEAK
**Research Design**	Randomized Control Trial or experimental study	Observational cohort or case-control studies	Uncontrolled studies
**Aims of the research:** Was the research objective clearly stated and directly related to review topic?	Clear description of objective. Outcome measures directly related to topic	Moderately clear, some details missing. Some outcome measures related to topic	Unclear or not stated
**Study population:** Was the study population clearly defined with inclusion/exclusion criteria stated and consistent?	Clear description of population and inclusion/exclusion criteria	Moderately clear, some details missing	Unclear or not stated
**Comparison population**: Was the comparison population selected from a comparable population in all respects?	Participants were comparable in at least: age, gender, weight, education, ethnicity, general or clinical population	Participants were comparable in at least three between: age, gender, weight, education, ethnicity, general or clinical population	No comparator group or unclear or not stated
**Sample size**	>100	50-99	<50
**Duration of follow-up**	1 year or greater follow-up	3 months to 11 months follow-up	< 3 months follow-up
**Description of the intervention**	Clear description of regimen and formula	Moderately clear, some details missing	Unclear or not stated
**Outcome parameters:** Were outcome parameters clearly defined? For outcomes that can vary, did the study clearly define different levels of the outcome?	Clear description, different levels of outcomes reported	Moderately clear description of the different levels of outcomes	No alternate level of outcome reported
**Data Collection Method:** Were scales and interviews assessing healthy and sustainable eating behaviors clearly stated and described?	Tools are valid and reliable	Tools are valid but reliability not described	No evidence of validity or reliability or not stated
**Measurement Bias**: Were the outcome measures clearly defined, valid, reliable and implemented consistently?	Valid, reliable, and explained in detail	Measurement valid but reliability not described	Self-reported by participants or not stated or unclear
**Selection Bias**: Is study sample representative of target population and if < 100% eligible cases were selected, were eligible cases randomized?	Very likely to be representative of target population, >80% participation rate<br>Eligible cases randomized for participation	Somewhat likely to be representative of target population,<br>60-79% participation<br>Eligible cases randomized for participation	< 60% participation rate or not stated<br>Eligible cases not randomized for participation
**Attrition Bias**: Was loss to follow-up after baseline minimized?	>80% follow-up after baseline	60-79% follow-up after baseline and explanation of those lost	< 60% follow-up after baseline or not reported
**Confounders**: Were key potential confounding variables measured and adjusted statistically for their impact on the relationship between intervention and outcome?	Confounders identified, discussed, and adjusted for statistically	Confounders identified and discussed	Unclear or not stated
**Single item scores: STRONG=3; MODERATE=2; WEAK=1; NON-AVAILABLE (N/A)=0**<br>**Overall Rating (0-25 = W; 26-30 = M; >31= S)**			

Customized checklist adapted from the National Institutes of Mental Health's tools (2021)

Supplementary Table S3. Risk of bias assessment and evaluation of the quality of the included studies

**CRITERIA**	Carfora et al. (2017)	Carfora & Catellani (2023)	Carfora et al. (2024)	De Croon et al. (2025)	Farias et al. (2023)	Flaherty et al. (2020)	Ghammachi et al. (2022)
**Research design**	S	S	S	W	S	W	W
**Aims of the research**	S	S	S	S	S	S	S
**Study population**	S	S	M	M	M	S	S
**Comparison population**	S	W	M	M	M	M	M
**Sample size**	S	S	S	S	W	W	W
**Duration of follow-up**	W	W	W	W	N/A	W	N/A
**Description of the intervention**	S	S	S	S	S	S	S
**Outcome parameters**	S	S	S	M	M	W	M
**Data collection method**	M	M	S	S	S	S	M
**Measurement bias**	S	S	S	S	S	W	M
**Selection Bias**	M	M	M	M	W	W	M
**Attrition Bias**	S	W	W	W	W	S	S
**Confounders**	W	W	S	W	W	W	W
Total score (S=3; M=2; W=1; N/A=0)	33	29	32	27	25	24	25
Overall Rating (0-25 = W; 26-30 = M; >31= S)	S	M	S	M	W	W	W

**CRITERIA**	Haas et al. (2022)	Herrewijn et al. (2021)	Monroe et al. (2015)	Meijers et al. (2022)	Plechatá et al. (2022a)	Plechatá et al. (2022b)	Wan et al. (2022)	Weber et al. (2021)
**Research design**	W	S	M	S	S	S	S	S
**Aims of the research**	S	S	S	S	S	S	S	S
**Study population**	M	S	S	S	S	S	S	S
**Comparison population**	M	M	S	S	M	S	S	M
**Sample size**	S	M	S	S	S	M	M	S
**Duration of follow-up**	N/A	N/A	N/A	W	W	N/A	N/A	N/A
**Description of the intervention**	M	S	S	S	S	S	S	S
**Outcome parameters**	S	S	S	S	S	S	S	S
**Data collection method**	S	S	S	S	S	S	W	S
**Measurement bias**	S	S	S	S	S	S	M	S
**Selection Bias**	W	W	S	W	M	S	S	M
**Attrition Bias**	N/A	N/A	S	S	S	W	N/A	N/A
**Confounders**	W	M	W	S	S	S	M	S
Total score (S=3; M=2; W=1; N/A=0)	24	28	33	35	35	33	28	31
Overall Rating (0-25 = W; 26-30 = M; >31= S)	W	M	S	S	S	S	M	S

Customized checklist adapted from the National Institutes of Mental Health's tools (2021)

Supplementary Table S4. PRISMA checklist

Section and Topic	Item #	Checklist item	Location where item is reported
TITLE			
**Title**	1	Identify the report as a systematic review.	Pag. 3
ABSTRACT			
**Abstract**	2	See the PRISMA 2020 for Abstracts checklist.	Pag. 1
INTRODUCTION			
**Rationale**	3	Describe the rationale for the review in the context of existing knowledge.	Pag. 2-3
**Objectives**	4	Provide an explicit statement of the objective(s) or question(s) the review addresses.	Pag. 3
METHODS			
**Eligibility criteria**	5	Specify the inclusion and exclusion criteria for the review and how studies were grouped for the syntheses.	Pag. 5
**Information sources**	6	Specify all databases, registers, websites, organisations, reference lists and other sources searched or consulted to identify studies. Specify the date when each source was last searched or consulted.	Pag. 3
**Search strategy**	7	Present the full search strategies for all databases, registers and websites, including any filters and limits used.	Pag. 3-5
**Selection process**	8	Specify the methods used to decide whether a study met the inclusion criteria of the review, including how many reviewers screened each record and each report retrieved, whether they worked independently, and if applicable, details of automation tools used in the process.	Pag. 4
**Data collection process**	9	Specify the methods used to collect data from reports, including how many reviewers collected data from each report, whether they worked independently, any processes for obtaining or confirming data from study investigators, and if applicable, details of automation tools used in the process.	Pag. 4
**Data items**	10a	List and define all outcomes for which data were sought. Specify whether all results that were compatible with each outcome domain in each study were sought (e.g. for all measures, time points, analyses), and if not, the methods used to decide which results to collect.	Pag. 5
	10b	List and define all other variables for which data were sought (e.g. participant and intervention characteristics, funding sources). Describe any assumptions made about any missing or unclear information.	Pag. 5, Table S1
**Study risk of bias assessment**	11	Specify the methods used to assess risk of bias in the included studies, including details of the tool(s) used, how many reviewers assessed each study and whether they worked independently, and if applicable, details of automation tools used in the process.	Pag. 7, Table S2
**Effect measures**	12	Specify for each outcome the effect measure(s) (e.g. risk ratio, mean difference) used in the synthesis or presentation of results.	N/A
**Synthesis methods**	13a	Describe the processes used to decide which studies were eligible for each synthesis (e.g. tabulating the study intervention characteristics and comparing against the planned groups for each synthesis (item #5)).	Pag. 4-6
	13b	Describe any methods required to prepare the data for presentation or synthesis, such as handling of missing summary statistics, or data conversions.	Pag. 4-6
	13c	Describe any methods used to tabulate or visually display results of individual studies and syntheses.	Pag. 4-7
	13d	Describe any methods used to synthesize results and provide a rationale for the choice(s). If meta-analysis was performed, describe the model(s), method(s) to identify the presence and extent of statistical heterogeneity, and software package(s) used.	N/A
	13e	Describe any methods used to explore possible causes of heterogeneity among study results (e.g. subgroup analysis, meta-regression).	N/A
	13f	Describe any sensitivity analyses conducted to assess robustness of the synthesized results.	N/A

**Reporting bias assessment**	14	Describe any methods used to assess risk of bias due to missing results in a synthesis (arising from reporting biases).	Pag. 7, Table S2
**Certainty assessment**	15	Describe any methods used to assess certainty (or confidence) in the body of evidence for an outcome.	N/A
RESULTS			
**Study selection**	16a	Describe the results of the search and selection process, from the number of records identified in the search to the number of studies included in the review, ideally using a flow diagram.	Pag. 6
	16b	Cite studies that might appear to meet the inclusion criteria, but which were excluded, and explain why they were excluded.	Pag. 6
**Study characteristics**	17	Cite each included study and present its characteristics.	Pag. 7-13, Table S1
**Risk of bias in studies**	18	Present assessments of risk of bias for each included study.	Table S2-S3
**Results of individual studies**	19	For all outcomes, present, for each study: (a) summary statistics for each group (where appropriate) and (b) an effect estimate and its precision (e.g. confidence/credible interval), ideally using structured tables or plots.	Pag. 7-13, Table S1
**Results of syntheses**	20a	For each synthesis, briefly summarise the characteristics and risk of bias among contributing studies.	Pag. 8, Table S3
	20b	Present results of all statistical syntheses conducted. If meta-analysis was done, present for each the summary estimate and its precision (e.g. confidence/credible interval) and measures of statistical heterogeneity. If comparing groups, describe the direction of the effect.	N/A
	20c	Present results of all investigations of possible causes of heterogeneity among study results.	Pag. 8-13
	20d	Present results of all sensitivity analyses conducted to assess the robustness of the synthesized results.	N/A
**Reporting biases**	21	Present assessments of risk of bias due to missing results (arising from reporting biases) for each synthesis assessed.	N/A
**Certainty of evidence**	22	Present assessments of certainty (or confidence) in the body of evidence for each outcome assessed.	N/A
DISCUSSION			
**Discussion**	23a	Provide a general interpretation of the results in the context of other evidence.	Pag. 14-16, 17
	23b	Discuss any limitations of the evidence included in the review.	Pag. 16
	23c	Discuss any limitations of the review processes used.	Pag. 16
	23d	Discuss implications of the results for practice, policy, and future research.	Pag. 16, 17
OTHER INFORMATION			
**Registration and protocol**	24a	Provide registration information for the review, including register name and registration number, or state that the review was not registered.	Pag. 4
	24b	Indicate where the review protocol can be accessed, or state that a protocol was not prepared.	Pag. 4
	24c	Describe and explain any amendments to information provided at registration or in the protocol.	N/A
**Support**	25	Describe sources of financial or non-financial support for the review, and the role of the funders or sponsors in the review.	Pag. 17
**Competing**	26	Declare any competing interests of review authors.	Pag. 17

**interests**			
**Availability of data, code and other materials**	27	Report which of the following are publicly available and where they can be found: template data collection forms; data extracted from included studies; data used for all analyses; analytic code; any other materials used in the review.	Pag. 17

From: Page MJ, McKenzie JE, Bossuyt PM, Boutron I, Hoffmann TC, Mulrow CD, et al. The PRISMA 2020 statement: an updated guideline for reporting systematic reviews. BMJ 2021;372:n71. doi: 10.1136/bmj.n71. This work is licensed under CC BY 4.0. To view a copy of this license, visit https://creativecommons.org/licenses/by/4.0/
